# A Comparison of Disease Severity and Outcomes in COVID-19 Cases Taking Angiotensin-Converting Enzyme Inhibitors/Angiotensin Receptor Blockers Versus Other Antihypertensive Drugs

**DOI:** 10.7759/cureus.12757

**Published:** 2021-01-18

**Authors:** Muhammad Abdullah, Khurshid Khan, Munaza Javed, Javeid Iqbal, Khalid Waheed, Muhammad Naeem Akhtar, Aijaz Zeeshan Khan Chachar

**Affiliations:** 1 Internal Medicine and Endocrinology, Fatima Memorial Hospital College of Medicine and Dentistry, Lahore, PAK; 2 Internal Medicine, University College of Medicine, the University of Lahore Teaching Hospital, Lahore, PAK; 3 Medicine, Fatima Memorial Hospital College of Medicine and Dentistry, Lahore, PAK; 4 Pulmonology, Lahore General Hospital, Lahore, PAK; 5 Internal Medicine, Fatima Memorial Hospital College of Medicine and Dentistry, Lahore, PAK

**Keywords:** covid-19, hypertension, aceis, arbs

## Abstract

Objective

In this study, we aimed to compare the severity and outcomes in hypertensive patients presenting with coronavirus disease 2019 (COVID-19) who were taking angiotensin-converting enzyme inhibitors (ACEIs)/angiotensin receptor blockers (ARBs) and those who were on other antihypertensive drugs.

Methods

This retrospective cohort study involved 182 hypertensive patients who presented with COVID-19 infection. The study population comprised 91 patients who were taking ACEIs/ARBs (group A) and 91 patients who were taking other antihypertensive drugs such as β-blockers (BBs), calcium channel blockers (CCBs), or thiazides (group B). All patients were provided the same type of treatment for the management of COVID-19. We recorded the data related to demographic and anthropometric variables as well as clinical symptoms during the treatment period. Disease severity and hospital mortality were the primary study endpoints.

Results

There was no significant difference in COVID-19-related outcomes between the groups except for the severity of lung infiltration on chest X-rays. There were 37 (41.1%) patients having >50% lung infiltration in group A and 53 (58.2%) in group B (p-value: 0.02). Severe disease was diagnosed in 37 (40.7%) patients in group A compared to 39 (42.7%) patients in group B (p-value: 0.76). In-hospital mortality was noted in 17 (18.7%) patients in group A and 22 (24.2%) patients in group B (p-value: 0.36).

Conclusion

Based on our results, we did not find any significant association between the use of ACEIs/ARBs and either the severity of COVID-19 infection necessitating admission to ICU or in-hospital mortality.

## Introduction

Coronavirus disease 2019 (COVID-19) was first reported in late 2019 from Wuhan, China, and it quickly spread all over the world within months, becoming a global pandemic. The disease is associated with severe respiratory compromise leading to high mortality rates, usually due to multi-organ failure [1]. Researchers have identified the risk factors that can potentially lead to increased susceptibility and severity of the disease and thereby worsen the outcomes in patients. Many recent studies conducted on COVID-19 patients have focused on comorbidities like cardiovascular disease (CVD), diabetes, and hypertension [2,3]. A meta-analysis of eight studies on the risk factors in COVID-19-infected patients has reported hypertension to be the most common comorbidity (17.1%), followed by CVD (16.4%) and diabetes (9.7%). The mortality rate in hypertensive patients was 6.0%, compared to 2.3% in patients without any of the above-mentioned morbidities [4].

The majority of hypertensive patients are prescribed angiotensin-converting enzyme inhibitors (ACEIs)/angiotensin receptor blockers (ARBs). Researches have reported that the use of these drugs causes the upregulation of ACE2 receptors (the main receptor of cell entry for COVID-19) and hence may increase vulnerability to COVID-19 infection and its severity [5,6]. So, in view of these findings, many researchers have raised concerns about the use of ACEIs and ARBs for the management of hypertension during the COVID-19 pandemic. However, data from some studies have reported no association between COVID-19 risk or its severity and patients taking these drugs [7,8]. This has led experts to conclude that there is insufficient data to support the discontinuation of ARBs and ACEIs during the COVID-19 pandemic, and they have called for more researches on the subject to reach more definitive conclusions. In light of this, we undertook the current study to compare the disease severity and outcomes in COVID-19 patients taking ACEIs/ARBs with those taking other antihypertensive drugs.

## Materials and methods

This retrospective cohort study involved 182 hypertensive patients who presented with COVID-19 infection to the department of medicine in three tertiary care hospitals in Lahore City, Pakistan. Hypertensive patients who have been under treatment for the condition for >6 months were included. Patients were divided into two groups: group A consisted of patients taking ACEIs/ARBs for their hypertension, and group B comprised hypertensive patients on medications other than ACEIs/ARBs. Patients with previous evidence of lung disease, e.g., those with asthma, chronic obstructive pulmonary disease (COPD), interstitial lung disease (ILD), or those having chronic renal or liver disease were excluded from the study. The data were collected from patients admitted within the last six months.

The study population comprised 91 patients who were taking ACEIs/ARBs (group A) and 91 patients who were taking other antihypertensive drugs such as β-blockers (BBs), calcium channel blockers (CCBs), or thiazides group A.

The data were retrieved retrospectively from medical records at the hospitals. Demographic and anthropometric variables, clinical symptoms during the treatment period, overall disease severity, and hospital mortality were recorded. Disease severity was determined according to the Pakistan guidelines on COVID-19, which were published in June 2020. Patients were considered to have severe disease if fever and cough were associated with the following conditions: respiratory rate of >30 breaths per minute, severe respiratory distress, oxygen saturation (SpO_2_) of ≤90% on room air, and chest X-rays showing >50% lung infiltration. The treatment regimen consisted of tocilizumab (as per indication), azithromycin, methylprednisolone, and enoxaparin for all patients.

Data analysis was conducted using the SPSS Statistics software version 25.0 (IBM, Armonk, NY). Independent samples t-test and chi-squared test were used for comparison of quantitative variables and qualitative variables respectively. A p-value of ≤0.05 was considered statistically significant.

## Results

There was no significant difference in terms of mean age, the duration of hypertension, and the presence of diabetes mellitus between the groups. There were 67 (73.6%) male patients in group A and 52 (57.1%) in group B (p-value: 0.02). There were more patients having ischemic heart disease (IHD) in group A: 30 (33%) versus 11 (12.1%) in group B (p-value: 0.001) (Table 1).

There was no significant difference in COVID-19-related outcomes between the groups except for the severity of lung infiltration on chest X-rays. There were 37 (41.1%) patients having >50% lung infiltration in group A and 53 (58.2%) in group B (p-value: 0.02). The mean length of hospital stay was 7.26 ±4.26 days in group A and 7.53 ±4.64 days in group B (p-value: 0.68). Severe disease was diagnosed in 37 (40.7%) patients in group A and 39 (42.7%) patients in group B (p-value: 0.76); the difference was not statistically significant. In-hospital mortality was noted in 17 (18.7%) patients in group A and 22 (24.2%) patients in group B (p-value: 0.36) (Table 2). 

We found a strong association between the duration of hypertension among patients and in-hospital mortality rates. The duration of hypertension was 12.89 ±9.26 years in patients who died versus 9.48 ±8.32 years in those who survived, with a regression coefficient value of 1.04 and p-value of 0.03 (Table 3).

**Table 1:** Comparison of baseline variables between the groups SD: standard deviation; IHD: ischemic heart disease

Variables	Group A	Group B	P-value
Age in years, mean ±SD	59.04 ±13.34	61.71 ±10.27	0.13
Gender			
Male, n (%)	67 (73.6%)	52 (57.1%)	0.02
Female, n (%)	24 (26.4%)	39 (42.9%)	
Duration of hypertension (years), mean ±SD	9.82 ±9.52	10.60 ±7.64	0.54
Diabetes mellitus, n (%)	53 (58.2%)	48 (52.7%)	0.45
IHD, n (%)	30 (33%)	11 (12.1%)	0.001

**Table 2:** Comparison of COVID-19-related variables between the groups COVID-19: coronavirus disease 2019; SD: standard deviation; CXR: chest X-ray; ICU: intensive care unit

Variables	Group A	Group B	P-value
Duration of COVID-19 infection (days), mean ±SD	5.26 ±2.10	5.60 ±2.66	0.34
Oxygen saturation (%), mean ±SD	84.4 ±14.44	83.13 ±14.10	0.54
Respiratory rate (breaths per minute), mean ±SD	30.27 ±13.11	31.28 ±10.05	0.56
Systolic blood pressure (mmHg), mean ±SD	128.03 ±22.64	125.71 ±21.43	0.47
>50% lung infiltration on CXR, n (%)	37 (41.1%)	53 (58.2%)	0.02
Overall disease severity, n (%)	37 (40.7%)	39 (42.9%)	0.76
ICU admission, n (%)	28 (30.8%)	38 (41.8%)	0.12
Hospital stay (days), mean ±SD	7.26 ±4.26	7.53 ±4.64	0.68
In-hospital mortality, n (%)	17 (18.7%)	22 (24.2%)	0.36

**Table 3:** Association between the duration of hypertension and in-hospital mortality HTN: hypertension; SD: standard deviation

	In-hospital mortality		Regression coefficient (β)	P-value
	Yes	No		
Duration of HTN (years), mean ±SD	12.89 ±9.26	9.48 ±8.32	1.04 (1.0-1.08)	0.03

## Discussion

The present study did not find any significant difference between patients taking ACEIs/ARBs and those taking other antihypertensive drugs in terms of disease severity and outcomes. The hypothesis that the use of ACEIs/ARBs can increase the chances of COVID-19 infection and its severity emerged from studies conducted on animals [9,10]. These studies reported that the ACE2 enzyme, a protein located on cell membranes, is used by severe acute respiratory syndrome coronavirus 2 (SARS-CoV-2) as a receptor to enter into the cells. However, studies have not reported any clear association between the upregulation of ACE2 and susceptibility and severity of COVID-19 infection [11-13]. The ACE2 is widely spread all over the body including alveoli, which is the main point of entry for SARS-CoV-2.

A recent review by Vaduganathan et al. analyzed the association between the use of ACEIs and COVID-19 and reported that there is insufficient data to determine whether effects on ACE2 receptors in humans caused by the use of ACEIs are similar to those found in animal studies. The results of the trials on the use of the renin-angiotensin-aldosterone system (RAAS) to determine whether the use of drugs such as valsartan or recombinant human ACE2 is beneficial in reducing the susceptibility and severity of symptoms have not been published yet. Moreover, the sudden withdrawal of ACEIs/ARBs, especially in high-risk patients such as those having heart failure or myocardial infarction, can cause hemodynamic instability or adverse health results. Hence, the authors recommended continuing the use of ACEIs/ARBs until sufficient evidence is available to justify the discontinuation of the use of ACEIs/ARBs in COVID-19 patients [14].

A study by Fosbøl et al. compared the outcomes in hypertensive patients with COVID-19 taking ACEIs/ARBs with those taking other antihypertensive drugs; the authors reported a mortality rate of 20.2% in patients taking ACEIs/ARBs and 8.3% in patients taking other drugs, while severe disease was diagnosed in 22.6% of patients taking ACEIs/ARBs compared to 10.4% of patients taking other drugs. However, after adjusting for age and gender, the mortality rate was found to be 9.4% in patients taking ACEIs/ARBs versus 9.7% in patients taking other drugs. Our findings contrast with these results and showed an in-hospital mortality of 18.7% in patients in group A and 24.2% in patients in group B (p-value: 0.36). Regarding disease severity, the authors concluded that the prior administration of ACEIs/ARBs did not increase the severity and mortality in hypertensive patients presenting with COVID-19 [15], which is highly comparable with our findings as our study also yielded no statistically significant difference in disease severity between group A (40.7%) and group B (42.7%) (p-value: 0.76).

Mehra et al. have also reported that the use of ACEIs had no association with an increase in COVID-19-related mortality [16], and their results are consistent with our study. A recent study by Hippisley-Cox et al. has reported that the use of ACEIs/ARBs significantly reduces the risk of acquiring COVID-19. They also reported that the use of ACEIs/ARBs did not increase the risk of admission to ICU [17], which aligns with our results, which also revealed that the prior use of ACEIs/ARBs did not escalate the risk of ICU transfer.

A study by Yan et al. has reported that the use of ACEIs can lead to a protective effect in COVID-19 patients. The authors reported a mortality rate of 19.4% in patients taking no antihypertensive drugs, 13.9% in those taking thiazides, 8.3% in those taking BBs, 5.8% in those taking CCBs, 8.3% in those taking ARBs, and 2.8% in those taking ACEIs. Severe disease was diagnosed in 14.8% of patients taking no drugs, 62.7% of those taking CCBs, 13.6% of those on BBs, 18.3% of those on ARBs, and only 5.3% of those taking ACEIs [18].

## Conclusions

The present study did not find any significant association between the use of ACEIs/ARBs and either the severity of COVID-19 infection necessitating admission to ICU or in-hospital mortality. Hence, our results discourage discontinuing the use of ACEIs/ARBs in hypertensive patients during the ongoing COVID-19 pandemic.
